# A mixed-methods study on the recruitment of patients from ethnic minority groups to clinical trials in a central London teaching hospital

**DOI:** 10.1016/j.conctc.2025.101475

**Published:** 2025-04-01

**Authors:** Cecilia Vindrola-Padros, Katie Gilchrist, Stuart Braverman, Rumana Omar, Edward Merivale, Ambar Hussenbux, Sanjay Khanna, Patience Renias-Zuva, Nick McNally, Rosamund Yu

**Affiliations:** a University College London (UCL), London, UK; b NIHR UCLH Biomedical Research Centre, University College London Hospitals NHS Foundation Trust, London, UK; c Roche Products Limited, London, UK

**Keywords:** Clinical trials, Ethnic minority groups, Recruitment, Inequalities

## Abstract

**Background/aims:**

Additional research is needed to fully understand barriers in recruitment to clinical trials and how these might affect different ethnic groups. The aim of this study was to explore the factors acting as barriers and facilitators in the process of recruiting patients to clinical trials in a UK (central London) teaching hospital, with a particular focus on patients from ethnic minority groups, and on areas where action could be taken.

**Methods:**

The study was designed as a mixed-methods study comprised of: 1) a quantitative workstream which explored variations in the ethnic and gender breakdown of people admitted to hospital relative to the demographic characteristics of patients enrolled into research studies at the hospital, and 2) a qualitative workstream which explored staff experiences of recruiting patients to clinical trials and patients’ experiences of being approached to take part in a clinical trial. The quantitative workstream provided the necessary context for the design of the qualitative workstream.

**Results:**

We found that the chances of being involved in research at the hospital were lower in all ethnic minority groups and lower amongst female patients. Some of the factors acting as barriers in trial participation included patients’ perceptions of clinical research as a form of experimental medicine that might have high risks, the role of family members in decision-making processes, and language barriers (where patients might not be fluent in English and the study information is not communicated in other languages). Potential strategies to address underrepresentation included the development of accessible information about research and how patient data are used, development of study materials in multiple languages and use of interpreters during the recruitment process, support for staff in involving family members in decision-making and a greater ethnic diversity within study teams.

**Conclusions:**

The under-representation of people from minority ethnic populations in clinical research remains a major challenge, impacting on the rigour and applicability of findings as well as implying some populations are missing out on the benefits of research. Study design needs to place greater emphasis on patient need and convenience and therefore to take greater account of the deterrent effect of financial and time burdens on trial participants. Recruiting sites and sponsors need to review the provision of interpreting and translation support for trials, including availability and cost to individual studies and staff confidence in reliability.

## 1 Introduction

The recruitment of patients to clinical trials is a process shaped not only by societal and historical factors but also by the organisational culture, history, infrastructure and priorities of healthcare organisations and clinical teams [[1], [2], [3]]. These factors influence which patients are approached to take part in clinical trials, when and how recruitment takes place, and the ultimate decision of patients [4,5]. Previous research has pointed to the lack of ethnic diversity in clinical trial participation [6,7], highlighting, as potential causes, limited trust in medical establishments, language barriers, clinicians’ limited cultural competence training and the increased burden trials put on the patient [[8], [9], [10], [11], [12], [13], [14], [15], [16]].

A systematic review exploring the factors acting as barriers and enablers in clinical trial participation in adult patients from ethnic minority groups [17] indicated that additional research is needed to fully understand barriers in recruitment and how these might affect different ethnic groups [17]. The review also called for more research to be carried out outside of the U.S. to reflect different healthcare systems and patient populations [17].

This study seeks to address this gap in the literature by exploring the factors acting as barriers and facilitators in the process of recruiting patients to clinical trials in a UK (central London) teaching hospital, with a particular focus on patients from ethnic minority groups, and on areas where action could be taken. In line with categories used in UK patient demographic data and the Office of National Statistics, the ethnic groups used were: White, Asian, Black, Other and Mixed. Following previous studies on this topic, we defined UK ethnic minority groups as groups other than the White group [18].

## 2 Methods

The study was designed as a mixed-methods study comprised of: 1) a quantitative workstream which explored variations in the ethnic and gender breakdown of people admitted to hospital relative to the demographic characteristics of patients enrolled into research studies at the hospital, and 2) a qualitative workstream which explored staff experiences of recruiting patients to clinical trials and patients' experiences of being approached to take part in a clinical trial. The quantitative workstream was designed to provide context regarding hospital activity for the qualitative study. The study was informed by a systematic review [17] and the study partners' desire to understand the impact of local trial recruitment processes. The study focused on four disease areas: breast cancer, rheumatology, stroke and Alzheimer's disease. In the light of feedback from public contributors and researchers and to gain a broader perspective, we extended the quantitative analysis to include a review of demographic data of admitted patients and trial participants with other cancers.

In order to identify specific areas where practical and actionable measures could be put in place, the study set out to answer the following research questions.

1. Does the data confirm a disparity between the proportion of research enrolments and the proportion of hospital admissions of patients from ethnic minority groups with the clinical conditions under consideration?
2. What are patients' and staff experiences of the trial recruitment process?
3. What are the main factors acting as barriers to recruiting patients from ethnic minority groups and challenges for gaining their consent?
4. What are the factors acting as facilitators for patients from ethnic minority groups' decision to take part in clinical research studies?

### 2.1 Data collection and analysis for the quantitative workstream

Data were obtained from NHS Digital comprising the number of hospital admissions between 2019–20 and 2021–22, where the HES Primary Diagnosis field (i.e. the main reason for admission) was one of the disease areas. Data sets were provided for this hospital's admissions specifically. These data sets were considered relative to the number of patients who have been actively enrolled into research studies at the hospital, where the study type matched one of these disease areas. The latter was extracted from the hospital's electronic health record system (Epic). In order to protect patient confidentiality, the data received from NHS Digital contained several lines of suppressed data. The research participation data featured numerous instances of uncoded ethnicity within the electronic health record.

Percentages and counts were used to summarise patients across gender, ethnicity and disease areas. Binomial regression was used to estimate the difference and ratio of percentages for patients enrolled in research studies in each ethnic group compared to the white group, with corresponding 95 % confidence intervals and overall P-values. It should be noted that NHS Digital did not provide figures where the total number of patients with a given combination of variables was less than 10, in order to preserve patient anonymity. In our main analyses, we have excluded these observations. We performed separate sensitivity analyses where the number of suppressed records were assumed to be 5 and 9. Data were analysed using the Stata software version 18 [19].

### 2.2 Data collection and analysis for the qualitative workstream

We carried out 41 interviews with 42 participants (18 with staff and 24 with patients/carers) across the disease areas. Of the staff interviewed, 10 were from ethnic minority groups and 8 from the White group. Of the patients/carers interviewed, 14 were from an ethnic minority group and 10 from the White group (see Table 1 in Appendix 2). Interviews were carried out via telephone or MS Teams using an interview topic guide (see Appendix 1) and were audio-recorded and transcribed. The topic guides were piloted in the first three interviews and the questions amended according to feedback. In two of the interviews with patients, an interpreter was used (the languages were Cantonese and Turkish). We were only able to interview 1 ethnic minority patient and carer in Alzheimer's disease due to the lack of ethnic minority patients to approach.

**Table 1:** Number and percentage of patients that comprise the hospital admissions and research cohorts.

Characteristics	hospital admissions n(%)	hospital research cohort n(%)
Gender		
Female	90,775 (53.44)	8,509 (41.76)
Male	79,085 (46.56)	11,844 (58.12)
Unknown	0 (0.0)	25 (0.12)
**Ethnic Group**		
**White**	94,000 (55.34)	7,854 (38.54)
**Black**	12,015 (7.07)	633 (3.11)
**Asian**	11,235 (6.61)	559 (2.74)
**Mixed**	3,270 (1.93)	185 (0.91)
**Other**	11,125 (6.55)	820 (4.02)
**Unknown**	38,215 (22.50)	10,327 (50.68)
**Disease Area**		
**Alzheimer's**	0 (0 %)	322 (1.58)
**Rheumatology**	27,210 (16.02)	740 (3.63)
**Stroke**	4,195 (2.47)	923 (4.53)
**Breast Cancer**	7,680 (4.52)	186 (0.91)
**Other Cancer**	130,775 (76.99)	18,207 (89.35)
**Total**	169,860	20,378

#### 2.2.1 Sampling and recruitment

Purposive sampling was used across the four disease areas to invite staff responsible for recruiting patients to clinical trials and patients who were eligible and asked to take part in research [20]. A sampling framework was followed to guide recruitment (Appendix 2). The process used to approach and consent patients for the interviews is described in Appendix 3.

#### 2.2.2 Analysis

Interview transcripts were analysed using framework analysis [21]. The transcripts were read for familiarisation and codes relevant to the research questions were identified by the team and added to a coding framework. One researcher (KG) coded the data and a second researcher cross-checked the coding (CVP). The coded data were charted following the case-by-theme approach used in framework analysis [21]. One researcher reviewed the framework to generate themes (KG) and a second researcher cross-checked the theme development (CVP).

### 2.3 Patient and public involvement and engagement (PPIE) and Steering Group

We engaged a panel of ethnic minority public contributors, supported by a manager with extensive PPIE experience. Public contributors reviewed the protocol, topic guides, participant information sheet and consent form prior to submission, advised on interim findings and reviewed this paper, providing written and verbal feedback. Two public contributors were full members of the Steering Group overseeing the conduct of the study for its duration. We provided a protected slot on each agenda to facilitate input from public contributors.

### 2.4 Ethical review and governance

The study was approved by the Health Research Authority (HRA) Wales REC 3 (IRAS: 318718). All participants took part voluntarily after providing informed and written consent. Consent was also confirmed verbally, prior to commencing the interview.

## 3 Results

### 3.1 Quantitative workstream

The results presented in Table 1, Table 2 treat the records that were suppressed by NHS Digital as missing. The sensitivity analyses did not change the conclusions, and so they are not reported in this paper. Table 1 presents the distribution of patients by gender and ethnicity. Overall figures are followed by a breakdown for each disease area. Comparisons are made between the hospital admissions and research cohort. We mainly describe the results based on the absolute differences in percentages, although relative differences in proportions are also presented.

**Table 2:** Comparison of research enrolment between males and females.

Sex	Overall total	Number enrolled in research (%)	Percentage difference (95 % Confidence Interval) in research enrolment	P-value	Ratio (95 % Confidence Interval) of enrolment proportions in research studies
Male	90,929	11,844 (13.0)	Comparator category	<0.001	
Female	99,284	8,509 (8.6)	4.4 (4.2 to 4.7)		0.7 (0.6 to 0.8)

**Table 3:** Overall comparison and comparison by clinical area for research enrolment of patients by ethnicity.

Clinical specialty	Ethnicity	Overall total	Number enrolled in research (%)	Percentage difference (95 %CI) in research enrolment	P-value	Ratio (95 % CI) of enrolment proportions in research studies
Overall	White	101,854	7,854 (7.7 %)	Comparator category	<0.001	Comparator category
	Black	12,648	633 (5.0 %)	2.7 (2.3–3.1)		0.6 (0.6 to 0.7)
	Asian	11,794	559 (4.7 %)	3.0 (2.6–3.4)		0.6 (0.6 to 0.7)
	Mixed	3,455	185 (5.4 %)	2.4 (1.6–3.1)		0.7 (0.6 to 0.8)
	Other	11,945	820 (6.9 %)	0.8 (0.4–1.3)		0.9 (0.8 to 1.0)
	Unknown	48,542	10,327 (21.3 %)	−13.6 (−14.0 to −13.2)		2.6 (2.7 to 2.8)
Rheumatology	White	17,206	441 (2.6 %)	Comparator category	<0.001	Comparator category
	Black	2,274	59 (2.6 %)	0.0 (−0.7 to 0.7)		1.0 (0.8 to 1.3)
	Asian	2,363	73 (3.1 %)	−0.5 (−1.3 to 0.2)		1.2 (0.9 to 1.5)
	Mixed	611	36 (5.9 %)	−3.3 (−5.2 to −1.4)		2.3 (1.6 to 3.2)
	Other	1,970	65 (3.3 %)	−0.7 (−1.6 to 0.1)		1.3 (1.0 to 1.7)
	Unknown	3,526	66 (1.9 %)	0.7 (0.2–1.2)		0.7 (0.6 to 0.9)
Stroke	White	2,804	609 (21.7 %)			
	Black	331	56 (16.9 %)	It was not possible to fit the model as there were no mixed ethnic group patients admitted for stroke.		
	Asian	236	31 (13.1 %)			
	Mixed	6	6 (100.0 %)			
	Other	904	169 (18.7 %)			
	Unknown	837	52 (6.2 %)			
Breast Cancer	White	4,586	106 (2.3 %)	Comparator category	0.185	Comparator category
	Black	472	22 (4.7 %)	−2.3 (−4.3 to −0.4)		2.0 (1.3 to 3.2)
	Asian	640	15 (2.3 %)	0.0 (−1.3 to 1.2)		1.0 (0.6 to 1.7)
	Mixed	164	4 (2.4 %)	−0.1 (−2.5 to 2.3)		1.0 (0.4 to 2.9)
	Other	840	15 (1.8 %)	0.5 (−0.5 to 1.5)		0.8 (0.4 to 1.3)
	Unknown	1,164	24 (2.1 %)	0.2 (−0.7 to 1.2)		0.9 (0.6 to 1.4)
Other Cancer	White	77,105	6,545 (8.5 %)	Comparator category	<0.001	Comparator category
	Black	9,564	489 (5.1 %)	3.4 (2.9 to 3.9)		0.6 (0.5 to 0.7)
	Asian	8,551	436 (5.1 %)	3.4 (2.9 to 3.9)		0.6 (0.5 to 0.7)
	Mixed	2,674	139 (5.2 %)	3.3 (2.4 to 4.2)		0.6 (0.5 to 0.7)
	Other	8,222	562 (6.8 %)	1.7 (1.1 to 2.2)		0.8 (0.7 to 0 0.9)
	Unknown	42,866	10,036 (23.4 %)	−14.9 (−15.4 to −14.5)		2.8 (2.7 to 2.8)
Alzheimers	White	153	153	It was not possible to fit the model as there were no hospital admissions where Alzheimers was the main reason for admission.		
	Black	7	7			
	Asian	4	4			
	Mixed	0	0			
	Other	9	9			
	Unknown	149	149			

Overall, females and those from all ethnic groups comprise a smaller percentage of the hospital research cohort than of the admissions cohort. The extent of under representation is lower in the White group compared to other known ethnic groups (Table 3). The research cohort has a considerably higher percentage of patients with missing ethnicity compared to that in the admissions. The distribution of patients across the disease areas are somewhat different between admissions and research cohorts, with the largest difference observed for Rheumatology. Other cancer is the most prevalent clinical area for both admission and the research cohorts.

The enrolment of patients in research studies is lower among females compared to that in males, with an absolute difference of 4.4 %.

The overall proportion of patients being enrolled in research studies across all disease areas is lower for all other known ethnic groups in comparison to the White group. The ‘Other’ group had the closest research enrollment percentage to White patients among ethnic minority groups. The other three identified ethnic groups had similar enrollment levels, though lower than that of White patients.

It was not possible to compare research enrolment across ethnic groups for Alzheimer's, as there were no admissions at the hospital where Alzheimer's was the main reason for inpatient admission. These patients would usually be managed in an outpatient setting. For Rheumatology, except for the Mixed group, there is little difference in the percentage of patients being enrolled in research studies in all the other known ethnic groups compared to that observed for the White group. White patients have the highest percentage of enrolment in research for stroke, followed by patients from the Other, Black and Asian groups. There are no patients from the Mixed group amongst those admitted with stroke in the hospital. The percentage of patients from the Black group enrolled in breast cancer research studies compared to patients from the White group is higher, although in absolute terms it is only 2.3 % higher, 95 % CI (0.4 % to 4.3 %). The differences in percentages in enrolment of patients from the Other group compared to patients from the White group are almost negligible for breast cancer. For Other Cancers, the percentage of patients enrolled in research studies is somewhat lower in all ethnic groups compared to that for the White group with the differences being very similar for Black, Asian and Mixed groups. However, the absolute differences are small.

The other cancer disease area also has the highest proportion of missing values for ethnicity in the research cohort. The percentage of patients with missing ethnicity values is small for the other disease areas. It should be noted that the P-values have not been adjusted for multiple significance testing and additionally these values may have been affected by the large sample sizes.

### 3.2 Qualitative workstream

The analysis of interview data was based on the creation of themes reflecting the factors acting as barriers and facilitators in the recruitment of patients to clinical research studies.

### 3.3 Factors acting as barriers in the recruitment of patients

In Table 4, we present the main factors acting as barriers in the recruitment of patients to clinical trials, sub-categories related to these factors and illustrative quotes from interviews with staff and patients.

**Table 4:** Factors acting as barriers in recruitment of patients.

Barriers affecting all ethnic groups		
Barrier Category	Sub-categories	Illustrative Quotes
**Study information**	Not patient-friendly	"Written very much in the style of a trial protocol. They're not written directed at patients" (Carer from the Alzheimer's disease service – White ethnic group).
	Patients had not seen posters	"Probably not, there's so many posters around the hospital anyway you don't really take them in to read. I don't recall seeing any about research but then I'm probably not looking for it" (Patient from the stroke service – ethnic minority).
**Access to patients**	Patient discharged	"Another group of patients that are not approached, the ones that are discharged before we are able to actually speak to them about the trial" (Staff from stroke service-White ethnic group).
	Patient away for appointments	"Busy with therapy sessions […] gone for scans" (Staff from stroke service- White ethnic group).
	Low numbers of ethnic minority patients in the service	"There's a certain population who end up being there. Usually, it's white, middle-class people. We haven't actually had anyone from an ethnic minority group in any of our trials that I can remember. Apart from maybe one or two" (Staff from Alzheimer's disease service- White ethnic group).
**Perception of the trial as experimental medicine**	Risk of taking trial drugs	"You do worry about the long-term side effects of drugs but as I said before, I couldn't see any way around that [ …]and they kept saying to me, everybody is different and nobody will react in the same way as somebody else" (Patient from Breast Cancer service – White ethnic group).
**Patient's health condition**	Fatigue	"Their general impairment, their fatigue" (Staff from stroke service-ethnic minority).
	Aphasia	"We have patients with aphasia, so this is a different barrier we face here" (Staff from stroke service-ethnic minority).
	Capacity	"A lot of the patients on the ward lack capacity so we have to wait for next of kin […] some patients don't have any or the next of kin only comes once a week" (Staff from stroke service-ethnic minority).
	Overwhelmed	“I think I would have found it quite stressful. Whether or not I'd said yes or no that would then probably have given me something to worry about which wouldn't have really been particularly helpful at that time" (Patient from stroke service – White ethnic group).
	Feeling unwell	"I might have said no once, just because that day I was feeling dizzy. As in that day, I for some reason when I went up to the clinic, I wasn't feeling well" (Patient from rheumatology service – ethnic minority).
**Burden produced by trial**	Too busy	"The time the patient actually has to spend after their clinic appointment, especially when consenting can be a longer process for some studies" (Staff from rheumatology service- White ethnic group).
	Additional visits needed	“Too many visits, they can't come so often” (Staff from breast cancer service-ethnic minority).
	Fitting in around work	"it's more about time now I think they need to go to work or if it's a study where they're having to be called again for it[ …]that's why it's really important to kind of match the appointments with their clinic appointments " (Staff from rheumatology service-ethnic minority).
	Travel and cost	“If someone is part of a community, that kind of stays very close to home and isn't used to, especially in a place like London coming into our site, it is quite the challenge in itself […] Some studies do require people to pay out of pocket in the first instance, and then they get reimbursed and people might not even be able to afford to pay out of pocket in the first instance” (Staff from Alzheimer's Disease service-ethnic minority).
	Additional procedures	"So, there's a definite feeling of should I just not bother and live my life, or am I prepared to commit myself to a lot of Procedures …” (Carer from Alzheimer's Disease service – White ethnic group).
**Concerns about their data**	Data protection or sharing data concerns	“If I thought that, you had the two pieces that I mentioned earlier, that the data and the identity, you had both pieces and I was feeling increasingly less confident of your ability to defend both of them simultaneously from, hack attempts that are ongoing constantly" (Patient from rheumatology service – ethnic minority).
	Don't want to share personal data	"Don't want to share their personal information" (Staff from stroke service-ethnic minority).
	Concerns around use of DNA	"The only other thing I think is the data protection issues and, yes. DNA sometimes makes people think twice" (Staff from rheumatology service- White ethnic group).
**Family influence**	Family impact on patient decision	"Sometimes they discuss with the family and yes as you mentioned, the family kind of have an effect on them" (Staff from stroke service-ethnic minority).
	Spouse answering questions on their behalf	“There's been families who have very much wanted to depend on their spouses, for example, to answer certain questions on their behalf.” (Staff from stroke service-ethnic minority).

**Barriers mentioned as specific to ethnic minority groups**		
**Barrier Category**	**Sub-categories**	**Illustrative Quotes**
**Language**	Language barrier impacted ethnic minority patients more than patients from White ethnic groups	"I guess you'd probably have a higher amount of people in ethnic minorities that we see in the clinic that don't speak English" (Staff from rheumatology service- White ethnic group).
	English language levels eligibility criteria	"Because of the nature of the type of studies we do, patients have to be fluent in English. That's because we monitor progress or effect of the drug based on its effect on your cognition and to do that, we need to do cognitive assessments in English, and it has to be standard across all the participants. So, it needs to be the same language" (Staff from Alzheimer's disease service- White ethnic group).
	Language barrier cannot always be overcome	“So, language is a barrier and, at times, we overcome it. At times despite our best efforts, we can't overcome it" (Staff from breast cancer service-ethnic minority).
	Inaccurate information in Electronic Healthcare Records	“When it said on epic that someone couldn't speak English or English wasn't their first language and needs an interpreter I found that very contradicting, to be honest, because when I approached them they understood things perfectly" (Staff from rheumatology service-ethnic minority).
	Access to interpreters	"I know language line [interpreting service] is available. I don't actually know if we can use it or not” (Staff from rheumatology service- White ethnic group).
	Accuracy of interpretation	"So even if you have an interpreter, they might just say yes or no to questions, and it doesn't really give you confidence that they have understood" (Staff from breast cancer service-ethnic minority).
	Many languages spoken in London	"London is really multicultural. So, it's just like what language would you choose" (Staff from stroke service-ethnic minority).
	Study documents not translated	"It was not translated to Chinese, it would have been easier to understand if it were in, Chinese because even if I ask somebody to take a look at the pamphlet and explain it to me, it's still very difficult for me to understand and then I would have to use Google translate to do the translation online but then, Google translate translation is not always correct" (Patient from breast cancer service – ethnic minority).
**Perception of the trial as ‘experimental medicine’**	Risk of taking trial drugs	"Some ethnic groups are not keen on taking investigational drugs" (Staff from Alzheimer's Disease service- White ethnic group).
**Family influence**	Male family members influencing female patient decisions	"Sometimes, especially with women, other family members are in charge of those decisions. That might be their son, it might be their husband, it might be their brother […] Which is not something we always see with our white population" (Staff from Alzheimer's Disease service- White ethnic group).
	Less open discussion amongst family members	"I think talking about the issues is problematic. I think the- being able to be open about their issues. I think sometimes it's more difficult for ethnic minorities, or just indeed families that don't discuss things like that between them" (Staff from Alzheimer's Disease service- White ethnic group).
	Large families – difficult to get consensus	"A lot of the time I think minorities, the patients might have capacity, but they always like, I want to get an opinion from their family member [ …]. A lot of them have come from very big, close-knit families. A patient might have many children and they all have different opinions, and they can't agree on one thing" (Staff from stroke service-ethnic minority).
	Family members more involved where there is a language barrier with patient	"I think sometimes certain, families will be very much more involved and opinionated as to whether, mum for example would go into the clinical trial. I'm not sure you get any more information as to why they do or don't, sometimes that's a language thing because it's the family that speak English" (Staff from breast cancer service- White ethnic group).
**Perceptions of research**	Patients unfamiliar with research	“Peoples' experiences of healthcare are different so that might then impact how trusting they are to certain treatments or research into further treatments because again, maybe a lack of understanding lack of knowledge, lack of experience because things are done differently in different places" (Patient from rheumatology service – ethnic minority).

#### 3.3.1 Communication - language barriers

Staff considered language barriers (defined as communication barriers where the staff and patient speak different languages) to be the biggest barrier in recruitment of patients to clinical research. While it was noted language barriers impacted patients of all ethnicities, staff highlighted that they impacted patients from ethnic minority backgrounds more than patients from White ethnic backgrounds. Language barriers were mentioned by all staff across all disease areas at numerous points throughout the interviews.

In breast cancer research, formal interpreters, family members or staff who spoke multiple languages did not always facilitate clear communication, meaning that some patients could not be recruited to a study. In Alzheimer's disease, research patients were required to have “fluent” levels of English to be eligible as cognitive tests needed to be delivered in English to reduce bias in result interpretation. Staff in rheumatology and stroke research mentioned inaccuracies in patients' Electronic Health Records regarding their level of English proficiency. These inaccuracies could limit the number of patients staff approached.

Staff members who used interpreters, either through NHS services or family members, expressed concern about knowing whether the information had been accurately interpreted and the validity of the patient's informed consent. The translation of written study related information also had its challenges as many languages are spoken in London and readily available translation into every language would require time and additional funding.

#### 3.3.2 Communication - study information

The study information was sometimes described as not being patient focused and full of technical information. There was no difference in opinion between ethnic groups regarding the communication of study related information. Most patients, regardless of ethnicity, reported that they had not seen or noticed posters about research before or after being invited to take part in a study and most did not know where to look for ongoing research studies.

#### 3.3.3 Perceptions of research

Staff suggested that lack of research awareness could be frequent in patients from ethnic minority backgrounds (particularly if they had lived in other countries before) and could contribute to doubts about participating in clinical research. Some staff felt patients were resistant if they perceived a study to be experimental. Patients from all ethnic groups discussed concerns about being part of a drug trial, mentioning the risk of trial drugs. Another concern raised frequently by patients was in relation to their data, mainly patients’ unwillingness to share personal data or worries about how the research team would protect their data.

#### 3.3.4 Capacity and access

Staff from the Alzheimer's disease service identified a major barrier to recruiting patients from ethnic minority backgrounds, observing that patients from ethnic minority groups were less likely to self-refer to the Alzheimer's disease service or be referred by a healthcare professional. This meant there were few ethnic minority patients to approach about research and reflects an inequity of healthcare access impacting on the diversity of research recruitment.

In stroke research, one of the barriers to recruitment for patients from all ethnic groups mentioned by staff was lack of mental capacity. This was amplified when the patient did not have family or friends nearby who could consent on their behalf. Another recruitment barrier specific to stroke and rheumatology research, but applicable to patients of all ethnicities, was patient availability. Patients could be discharged from hospital or away having tests before the recruitment team could approach them about a study.

#### 3.3.5 Study burden and the patient's health condition

The burden of study activities acted as a barrier to recruitment for all ethnic groups, particularly the length and intensity of studies, and travel and cost involved. Patients said decisions to take part in research often involved weighing the potential benefits of taking part against the extra hospital visits needed when feeling fatigued, unwell or overwhelmed.

### 3.4 The role of family members

The role family members could play in patients' decisions to take part in research was mentioned frequently in the staff interviews and was highlighted as a factor that could be more relevant in the decision-making process of patients from ethnic minority groups (which groups were not specified). Staff said families with ethnic minority backgrounds might rely on male family members to make decisions about research participation, influencing a female patient's decision. Furthermore, larger families could involve more people in decision-making, often on stigmatised and difficult to discuss topics, ultimately resulting in decisions that might not reflect the patient's preference. Family members' reasons for refusal to take part in clinical trials were similar to those of patients, mainly the belief that the research could be experimental and the desire to reduce patient burden.

#### 3.4.1 Factors acting as facilitators in the recruitment of patients

In Table 5, we present the main factors acting as facilitators in the recruitment of patients to clinical research studies, sub-categories related to these factors and illustrative quotes from interviews with staff and patients.

**Table 5:** Factors acting as facilitators in the recruitment of patients to clinical research.

Facilitators affecting all ethnic groups		
Facilitator Category	Sub-categories	Illustrative Quotes
**Quality and length of life**	Deescalate treatments	"A lot of clinical trials or clinical research in early breast cancer is also looking at means of deescalating treatments, so giving less chemotherapy based on biomarkers and so on and there's a lot of motivation from patients to participate in that as well" (Patient from breast cancer service – White ethnic group).
	Prolong life	"I just felt that I would rather have a little bit more life, than not and I was prepared to take the risk" (Patient from breast cancer service – ethnic minority).
	Maintain quality of life	"The hope that it will maintain his quality of life” (Carer from Alzheimer's disease service – White ethnic group).
**Altruism**	Improve treatment	“I just said yes, anything you can do that would make treatment safer and more available would be a good thing" (Patient from stroke service – White ethnic group).
	Help their children (specific to hereditary conditions)	"It's also going to potentially impact our children, so you know, there's kind of like a personal hope that it's going to affect you, but also from my side contributing towards the children" (Carer from Alzheimer's disease service – White ethnic group).
	Help future patients	"Nice feeling, you know, that yes, I'd contributed towards an answer that might relieve a lot of pain" (Patient from rheumatology service – ethnic minority).
	Contribute to research	"I'm very happy to help medical research and to contribute to science's greater knowledge and understanding" (Patient from stroke service – White ethnic group).
**Access to treatment**	Lack of treatment options	"Some groups of patients who have rare diseases for which we don't have any treatment or good treatment and they're more likely to say yes to participating in any trial" (Patient from rheumatology service – White ethnic group).

**Facilitators specific to ethnic minority groups**		
**Facilitator Category**	**Sub-categories**	**Illustrative Quotes**
**Language**	Assessing language levels	"Sometimes on Epic, it will say next to the patient's name if they require a translator but it's not always accurate, so we normally just check in the notes or check with the team if the person speaks English" (Staff from stroke service- White ethnic group).
	Using interpreters (professional, staff or family)	"Sometimes if the patient speaks the same language as a member of the team that can be useful" (Staff from stroke service-ethnic minority).
	Translated study documents	"If you do have translated versions of documents, yes, there is at least better understanding of the treatments and the study that's being all research that's being offered" (Staff from breast cancer service-ethnic minority).
**Ethnic representation within research teams**	Ethnic diversity within research teams	"One of my colleagues who was from Nigerian ethnicity was the main recruiter so I think that helped that, yes, it wasn't just a white research nurse" (Staff from rheumatology service- White ethnic group).
**Perceptions of research**	Understanding research	“I think what I try and do is try and understand the culture more and try and relate to it more with them[ …]I think in the beginning sometimes has been quite important and perhaps where I felt that someone would have not been interested. Suddenly then starts to warm up to the idea of the research and really understand it" (Staff from stroke service-ethnic minority).

#### 3.4.2 Motivation for taking part

When they were asked about their motivation for taking part in clinical research studies, patients mentioned to: help others; increase their quality of life; prolong life reduce symptoms; deescalate treatments while maintaining the same benefits; and find treatments not available as standard care. We did not find clear trends in differences regarding the motivations for participating in research across ethnic groups.

#### 3.4.3 Addressing language barriers

When staff were asked about the factors that they thought facilitated recruitment, they mentioned the use of formal interpreters or staff who spoke different languages and the use of translated study documents to overcome communication challenges. Staff also mentioned not relying on the information they found in the patients’ electronic healthcare record and assessing the level of English proficiency themselves. Some staff members also mentioned that ethnic diversity within the research team made patients from ethnic minority groups more willing to discuss clinical research.

## 4 Discussion

In this mixed-methods study, we found that the chances of being involved in research at the hospital were lower in all ethnic minority groups and lower amongst female patients. This underrepresentation could be explained not only by societal factors but also by patients’ perceptions of clinical research as a form of experimental medicine that might have high risks, the role of family members in decision-making processes, and language barriers (where patients might not be fluent in English and the study information is not communicated in other languages). Potential strategies to address underrepresentation include development of accessible information about research and how patient data is used, development of study materials in multiple languages and use of interpreters during the recruitment process, support for staff in involving family members in decision-making and a greater ethnic diversity within study teams.

Our study supports previous research showing that patients from ethnic minority groups might decline study participation due to the additional burden placed by the study (i.e. extra hospital visits and procedures) [22], the inability to understand study information (due to language barriers or inaccessible trial materials) [23], a lack of trust in clinical teams and healthcare organisations [2,24] and the limited cultural diversity and cultural competence of clinical teams [9,16,25]. Even though most of this published evidence has emerged from the US, our study has shown that similar factors act as barriers in UK patient populations.

When considering decision-making in relation to trial participation, our study has pointed to the role played by family members in this process and the need to communicate and involve people additionally to the patient. As Albrecht and colleagues [26] argued in their study on communication in the context of clinical trials, trial participation should be seen as an alliance that involves the doctor, patient and family companions. Our study also pointed to inaccuracies in the recording of the patient's level of English proficiency in electronic health records, confirming the findings of other studies pointing to variability in the degree of accuracy of preferred language fields in electronic health records [27]. Access to interpreting services and translated materials was also identified as a barrier and is consistent with other research on barriers in the delivery of interpreting services in the NHS [28]. This study has highlighted that the inequity of access to healthcare that different ethnic groups face can be a key factor in the under-representation of ethnic minority patients in clinical research.

### 4.1 Limitations of the study

The findings of this study should be considered in light of its limitations. In the qualitative workstream, the recruitment of patients from ethnic minority groups proved difficult and the study did not meet the numbers of patients originally included in our sampling framework for the stroke and Alzheimer's disease areas. Research teams helping with recruitment found it difficult to facilitate the recruitment of patients from ethnic minority groups, even though this was the main purpose of this study. Research teams also reported limited time available to dedicate to recruiting to the project. In Alzheimer's disease, the lack of ethnic minority patients to recruit to this study, was a major limitation. These recruitment challenges provided important lessons for future studies including the need to provide simpler patient recruitment materials available in multiple languages, simple access to interpreters for remote interviews and more regular engagement with clinical teams identifying potential participants. Another key limitation was that it was not possible to interview patients who had been approached about a trial and had declined.

In the quantitative workstream, the main caveats pertained to uncoded or suppressed data around patient ethnicity. Overall, 50 % of patients who had been actively enrolled onto a research study at the hospital did not have their ethnicity coded in their electronic medical record. There was clear variability in data completeness across disease areas, with ‘other cancer’ having a marked influence on this overall figure, given that ethnicity data were missing for 55 % of patients and it accounted for 89 % of the overall research cohort.

The hospital admission data set contained a far smaller percentage of missing data (1 %). However, it did contain the added complication of data suppression, where the number of patients meeting a specific combination of criteria was between 1 and 10. Data were suppressed by NHS Digital to avoid identifying individuals. Suppressed data impacted between 0.01 % and 1.5 % of the hospital admission total.

Whilst attempts were made to mitigate the risk of bias, the high degree of missingness means that conclusions should be treated with caution. It should also be noted that the two data sets (research enrolments + hospital admissions) may not be directly comparable as the majority of hospital research contacts take place in an outpatient setting. This was most notable for Alzheimer's Disease, where there were no inpatient admissions, preventing comparison with the research cohort. Nonetheless, hospital admission data was chosen as the comparator because NHS Digital advised that ethnicity data would be more complete for inpatient admissions because NHS Trusts are mandated to provide this information. The COVID-19 pandemic could have also impacted on the recruitment of patients in non-COVID trials.

This study focussed only on exploring the factors acting as barriers and facilitators in the process of recruiting patients to clinical research studies and did not explore broader inequalities that exist in accessing healthcare. A potential indication that significant barriers exist in accessing and navigating through the healthcare system is the lack of ethnic minority patients available to interview in the Alzheimer's disease clinic. There are added complexities for these patients when being diagnosed with complex referral pathways through primary care and memory clinics and varying reasons for admissions secondary to Alzheimer's disease.

It is also important to consider that this study took place in a large London teaching hospital with a diverse patient population and access to research sources that might not be found in other areas of the country.

## 5 Conclusions and implications of the study

The under-representation of people from minority ethnic populations in clinical research remains a major challenge, impacting on the rigour and applicability of findings as well as implying some populations are missing out on the benefits of research. In this study, we found that the chances of being involved in research at the hospital were lower in all ethnic minority groups and lower amongst female patients. The study shed light on some of the barriers and facilitators of the recruitment of ethnic minority patients to clinical research within the UK.

The study aimed to identify factors within the recruitment process that act as barriers to the recruitment of ethnic minority participants, for which practical and actionable solutions could be found. However, many barriers reflect societal and historical factors. Many barriers are also a result of broader health inequalities that require structural changes that cannot be addressed by individual study designs. Researchers, sponsors and recruiting sites need to raise awareness of these issues and work to find possible sustainable solutions.

One of the main contributions of this study, is a range of actions that can be implemented to address a fundamental limitation of clinical research in the current context, including exploring the role of artificial intelligence and other emerging technological solutions. Recruiting sites and sponsors need to review the provision of interpreting and translation support for trials, including availability and cost to individual studies and staff confidence in reliability. Accessibility and cost should be key factors in study design.

The recording on the patient record of proficiency in English language and any staff assumptions about language proficiency need to be highlighted as issues when setting up studies and would benefit from further investigation. We recommend the development of better recording of participant demographics. Addressing many of the barriers identified will require raising awareness of the barriers and we recommend putting in place targeted and proactive training and support for recruiting staff. This would need to include empowering staff to approach and intentionally engage with ethnic minority patients and to effectively involve families and friends in decision-making.

Study design needs to place greater emphasis on patient need and convenience and therefore to take greater account of the deterrent effect of financial and time burdens on trial participants. It is recommended that further investigation is needed into the deterrent effect of these burdens specifically on underserved groups. We recommend sponsors and recruiting sites follow the guidance of the Health Research Authority (HRA) report People-Centred Clinical Research.

Study design should also take account of the deterrent effect of any previous negative healthcare experiences of patients. Much work is already being carried out to increase confidence in clinical research among different population groups. However, this work must not overlook the issue of trust specifically in the use of data in clinical research, which should be addressed in communications about studies and research in general.

We recommend further rigorous investigation into two major areas highlighted by this study: the role of the family in patients’ decision whether or not to take part in research and the impact of referral patterns and access to specialist health services on the availability of diverse potential participants.

When designing trials sponsors are recommended to proactively engage with specific ethnic minority patient communities and to mandate diversity and inclusion. It is vital that engagement with different communities is sustained and impactful. We also recommend greater consideration is given to the use of dedicated research roles to address any capacity issues, and to building up engagement and trust between patients and clinicians within the research environment.

Sponsors, including commercial sponsors, and UK regulators such as the Medicines & Healthcare products Regulatory Agency (MHRA) and the HRA need to work collaboratively with the NHS and Voluntary and Community Sector organisations to implement wider systemic change to address research recruitment barriers for ethnic minority patient communities and bring equity in clinical trial recruitment representation.

## CRediT authorship contribution statement

**Cecilia Vindrola-Padros:** Writing – review & editing, Writing – original draft, Supervision, Methodology, Funding acquisition, Formal analysis, Conceptualization. **Katie Gilchrist:** Writing – review & editing, Writing – original draft, Methodology, Formal analysis, Data curation. **Stuart Braverman:** Writing – review & editing, Writing – original draft, Methodology, Formal analysis, Data curation, Conceptualization. **Rumana Omar:** Writing – review & editing, Writing – original draft, Validation, Formal analysis. **Edward Merivale:** Writing – review & editing, Writing – original draft, Methodology, Funding acquisition, Conceptualization. **Ambar Hussenbux:** Writing – review & editing, Writing – original draft, Methodology, Funding acquisition, Conceptualization. **Sanjay Khanna:** Writing – review & editing, Writing – original draft. **Patience Renias-Zuva:** Writing – review & editing, Writing – original draft, Methodology, Conceptualization. **Nick McNally:** Writing – review & editing, Methodology, Funding acquisition. **Rosamund Yu:** Writing – review & editing, Writing – original draft, Project administration, Methodology, Funding acquisition, Conceptualization.

## Disclaimer

This research was completed as part of a collaborative working agreement between University College London Hospitals NHS Foundation trust (UCLH, referred to as ‘the hospital’) and Roche Products Limited (Roche). Both UCLH and Roche were involved in the development and completion of this project. The National Institute for Health and Care Research University College London Hospitals Biomedical Research Centre provided infrastructure support for this work including salary support for several of the contributors.

UCLH and Roche stress that although this study begins to map out and identify specific barriers to the recruitment of ethnic minority patients to research in the UK, these barriers may not apply to all ethnic minority groups and different cultural nuances require deeper contextualisation. Many barriers are the result of societal and historical factors that this study does not include. This study focuses on what happens during the recruitment process, with a view to identifying practical and actionable measures that sponsors and the NHS can implement.

## Declaration of competing interest

The authors declare the following financial interests/personal relationships which may be considered as potential competing interests:This research was completed as part of a collaborative working agreement between University College London Hospitals NHS Foundation trust (UCLH, referred to as ‘the hospital’) and Roche Products Limited (Roche). Both UCLH and Roche were involved in the development and completion of this project.

## Data Availability

The authors do not have permission to share data.
