# Supplementary material for: A mixed-methods study on the recruitment of patients from ethnic minority groups to clinical trials in a central London teaching hospital
Source: Contemp Clin Trials Commun. 2025 Apr 1;45:101475. doi: 10.1016/j.conctc.2025.101475 (PMC12002992; doi:10.1016/j.conctc.2025.101475)
Supplement: Multimedia component 1 [file mmc1.docx]

**Appendix 1.** Topic guides

**Staff topic guide**

***Self-reporting characteristics***

How do you describe your ethnicity:

Please may we know your age:

What gender do you identify as:

Location of employment:

Employment position:

***Interviewee role***

1. I was wondering if we could begin with a description of your current role.
2. Could you tell me about any clinical trial recruitment you have been involved in?
   1. In what capacity, how many, over what period, conditions/interventions

***INCLUDE ethnicity Framework mapping (based on the four key questions)***

1. Before recruitment begins, how do you ensure you are going to approach relevant people for who the trial results are intended? (which groups would benefit from the intervention if effective)
2. How do you identify and respond to scenarios where some groups may respond to the intervention in different ways? For example certain ethnic groups
3. How do you identify and respond to scenarios where the intervention, or delivery of, may make it harder for some groups to engage with the trial? Are there certain ethnic groups which may find it harder to engage with the trial?
4. In your experience are there certain scenarios? (eligibility, recruitment/consent process) where the design of the trial may make it harder for ethnic minority groups to engage with the trial? How do you identify and respond to this kind of scenarios?

***Trial recruitment process***

1. How are patients identified to be approached?
   1. Are there any reasons why certain patients are or are not approached? Do any of these reasons particularly affect ethnic minority patients?
2. In your opinion, do you feel the ethnicity of a participant shapes the way in which recruiters’ approach and invite them to participate in a clinical trial?

Notes:

1. How are patients asked to participate in trial? Are there any reasons why patients are not asked? And do any of those reasons particularly apply to ethnic minority patients?

Notes:

1. How do you approach potential participants where English is not their first language?

Notes:

1. Are they required to have a certain level of English speaking to take part in a trial?
2. Do you offer and arrange interpreters?
3. If so, how is correct translation ensured?

Notes:

1. How do you approach participants who may require other types of interpreters?
   1. Do you offer and arrange a sign language service for those who are deaf or hard of hearing?
   2. Are there times where family members need to help explain what you are asking?
2. What happens during the recruitment process?
   1. What are the steps, how long does it take?
   2. Are there any particular processes or systems you use?
   3. Do you think any of those processes impact on the recruitment of ethnic minority patients?
3. Have you attended any training at CENTRAL LONDON TEACHING HOSPITAL HOSPITAL based on communicating with different groups of people or people of different ethnicities? (If not at CENTRAL LONDON TEACHING HOSPITAL HOSPITAL, anywhere else?)
   1. Unconscious bias training? NIHR INCLUDE Training
4. Are there any reflective practices following a trial to improve future trial recruitment?

***Trial consent***

1. What are the main barriers to consenting?

Notes:

1. Why have patients refused participation in the trial?
   1. Where patients have refused do they ask many questions, consult with family?
   2. Is there any process for asking their reasons for refusal? (*understand this may be tricky to ask as they don’t need to give a reason*)
   3. Are any reasons more common with certain ethnic backgrounds?
2. Why have patients consented to take part in the trial?

Notes:

1. Where patients have consented, how long has this decision taken?
   1. Do they ask many questions, consult with family?
   2. Is ethnic background a factor in that decision? Do people from ethnic minority groups have a particular motivation to consent compared with other groups?

***Participant retention***

1. Why do patients decide to withdraw from the trial?

Notes:

1. What do you think could improve the retention of participants?
2. Anything else specifically for ethnic minority groups?

**Patient topic guide**

***Self-reporting characteristics***

How do you describe your ethnicity:

Please may we know your age:

What gender do you identify as:

Where were you treated?

Location (London or outside of London):

***Section 1 Trial recruitment process***

1. Have you ever taken part in a clinical trial? (clinical trial)
   (If no, move to section 2)
2. (If yes) Could you tell me about your personal experience of taking part in such trials?
3. How many have you been invited to and how many did you participate in?
4. What were the clinical trials for? Which health conditions or treatments were they for?
5. Do you know what phase the trial was?
6. What stage/severity was your condition at when you took part in the trial?
7. Are you aware of any trials that you felt may have been relevant to your condition that you were not asked to participate in?
   - 1. If so, are there any reasons why you think you may not have been asked?
8. Have you asked your treating physician if you can be part of a trial that you thought was relevant to your condition, but your treating physician didn’t think was relevant?
9. Do you know where to search for trials that are currently ongoing or potential trials that will be recruiting? (*here, we can also listen out for community engagement/collaboration in research*)
10. Thinking about the trials you were asked to participate in, how were you approached / asked to be involved?
11. Who made the approach to you about the trial? (*professional group, gender, ethnicity, anything about them that influenced decision*)
12. Where did the approach take place? (*clinical setting, privacy of consulting room, busy noisy waiting room, other people around, hospital ward, community setting*)
13. What was the context of approach? (*during/end of appointment, part of other conversations, how did they introduce it*)
14. Had the person who approached you, been involved in your care at any time up to that point? Did you already know them?
15. Were your family present? Did they, or anyone else influence your decision?
16. Had you seen posters / other adverts? Or were you made aware of the trial before being approached – did this make a difference or do you feel this would have made a difference to your participation?
    1. If saw posters, ask where
17. Did you feel these approaches were appropriate?
18. How did you find the approaches?
19. Were you glad to be approached?
20. If not, why?
21. How do you feel any initial approach could have been better?
22. Thinking about your own interactions with staff, Were they friendly, approachable?
    1. Did they provide information in a way you could access / understand it?
    2. Were they able to answer your questions or find the answers for you?
    3. Did you get answers to the questions you asked in an understandable manner?
    4. Were you able to easily contact staff if you needed to using the contact details provided to you?
23. For the trials you consented to take part in, would you mind sharing with us why this was?
24. What motivated you to want to take part in a trial?
25. What did you find helpful or unhelpful about the process?
26. Information accessible?
27. Language and written format layout easy to understand?
28. Who asked
29. How they asked
30. Did they provide clarity on why trials could be an alternative treatment option? (*the benefits of research*)
31. The information they told you (*about the trial/about the disease*)
32. Any financial costs? (travel, accommodation, food)
33. How did research teams make the trial, you participated in, accessible/appealing? (*Here we can listen out for flexibility/childcare*)
34. Were you given plenty of time and opportunity to ask questions about the trial did you have time to talk to family and friends – did this make a difference?
35. If you saw posters or were aware beforehand, did this help you with any questions you had?
36. Is there anything else you would like to share with us about your decision to participate?

**Section 2 Participant did not take part in trial**

1. You haven’t taken part in a clinical trial, could you tell me about your experiences of being asked to take part in a clinical trial?
2. Where you declined to participate, it would be helpful if you could tell us the reasons why you chose not to take part in the trial?
3. Was there anything specific that influenced your decision or put you off taking part?
4. At what stage of the recruitment process did you decide you didn’t want to take part in the trial? Was this the same stage you told the trial team that you wanted to withdraw? (*to establish if they delayed voicing that they didn’t want to take part*)
5. Is there anything that could have been done differently that would not have put you off, or may have encouraged you to participate in the trial?
6. Different approach
7. Different way of talking to you
8. Different person approaching you
9. Different place of approach or trial
10. What you were told / information you were given
11. More accessible information
12. Use of language (was it difficult to understand)
13. Were there any financial costs that impacted your decision? (travel, accommodation, food)
14. How can research teams make trial participation more accessible/appealing?
15. Do you feel you were given plenty of time and opportunity to ask questions about the trial?
16. Were you able to talk to family or friends and did they help you decide or influence your decision?
17. Had you see posters / other adverts? Were you aware before you were approached – did this make a difference (*ask in context of having time to ask questions*)
    1. If saw posters, ask where
18. If not, do you think this impacted your decision not to take part?
19. Is there anything else you would like to share with us about your decision not to participate?

***Section 3 Participant retention***

1. If you participated, did you take part in the trial until the end?
2. If no, at what stage did you decide you no longer wanted to take part? Was this the same stage you actually withdrew? (to establish if they delayed voicing that they didn’t want to continue)
3. If no, it would be helpful if you could explain the reasons you decided to withdraw (medical or personal)
4. If **no**, is there anything that would have encouraged you to continue?
5. Did you feel comfortable choosing to withdraw from the trial?
6. If **yes**, can you tell us about anything that encouraged you to stay involved?
7. Did you feel comfortable continuing in the trial?
8. Did you feel you could withdraw if you wanted to and that doing so wouldn’t impact ongoing treatment or care?

**Appendix 2.** Sampling framework and characteristics table

Table 1: Sampling framework

| **Participant** | **Target number of interviews**  **20 patients/carers and 20 staff** |
| --- | --- |
| Breast cancer clinic (patients/carers) | 5 (at least 3 of an ethnic minority) |
| Breast cancer clinic (staff) | 5 (at least 1 of an ethnic minority) |
| Alzheimer's Disease clinic (patients/carers) | 5 (at least 3 of an ethnic minority) |
| Alzheimer’s Disease clinic (staff) | 5 (at least 1 of an ethnic minority) |
| Stroke clinic (patients/carers) | 5 (at least 3 of an ethnic minority) |
| Stroke clinic (staff) | 5 (at least 1 of an ethnic minority) |
| Rheumatology clinic (patients/carers) | 5 (at least 3 of an ethnic minority) |
| Rheumatology clinic (staff) | 5 (at least 1 of an ethnic minority) |
| **TOTAL** | **40** |

Table 2: Characteristics of interview participants

| ***Ethnic group*** | **Staff** | **Patients/carers** |  |  |
| --- | --- | --- | --- | --- |
| Participants from ethnic minority groups | 10 | 14 |  |  |
| Participants from White group | 8 | 10 |  |  |
| ***Total*** | **18** | **24** |  |  |
| ***Clinical area*** | **Staff** | **Patients/carers** | **Patients from ethnic minority groups** | **Patients from White group** |
| Alzheimer's disease | 4 | 4 | 1 | 4 |
| Stroke | 5 | 6 | 2 | 4 |
| Rheumatology | 5 | 8 | 7 | 1 |
| Breast cancer | 4 | 5 | 4 | 1 |
| ***Total*** | **18** | **24** | **14** | **10** |
| ***Type of study invited to*** |  |  |  |  |
| *Clinical trial (drug study)* | N/A | 11 |  |  |
| *Observational study* | N/A | 12 |  |  |
| *Register to be contacted about studies* | N/A | 1 |  |  |
| ***Total*** | **18** | **24** |  |  |
| ***Participant age*** | **Staff** | **Patients/carers** |  |  |
| 20-29 yrs | 2 | 0 |  |  |
| 30-39 yrs | 8 | 4 |  |  |
| 40-49 yrs | 4 | 4 |  |  |
| 50-59 yrs | 3 | 7 |  |  |
| 60-69 yrs | 1 | 4 |  |  |
| 70-79 yrs | 0 | 5 |  |  |
| ***Total*** | **18** | **24** |  |  |
| ***Gender*** | **Staff** | **Patients/carers** |  |  |
| Male | 3 | 7 |  |  |
| Female | 15 | 17 |  |  |
| ***Total*** | **18** | **24** |  |  |

**Appendix 3.** Recruitment process of interviews with staff and patients/carers

The research team held meetings with clinical leads from each disease area to establish the process for recruitment into this study. Each clinical lead advertised the present study to their trial recruitment staff in team meetings, providing them with the UCL researcher contact details so that they could enquire anonymously. Patients were initially approached by clinicians within their routine appointments and were asked for verbal consent for the UCL researcher to contact them with further information about the study. Both staff and patients received two follow-up reminders of the study. Potential participants were sent a participant information sheet and consent form, by the UCL researcher, and were provided with the opportunity to ask any questions. Once participants were satisfied all their questions about the study were answered and they decided they wanted to take part, they offered consent for participation to the UCL researcher, and an interview was arranged at a time to suit the participant. When participants met the UCL researcher for the interview, they were reminded that it was voluntary and that they may withdraw at any time or skip any questions. Participants were advised that an audio recording of the interview would be taken, and verbal consent was obtained prior to commencing.

**Appendix 4. Comparison of % of hospital admissions by ethnic groups with % of trial participants by ethnic groups**

**
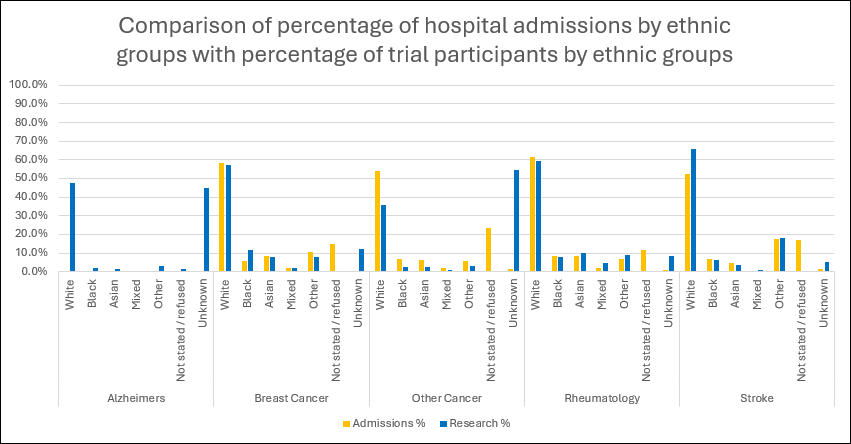
**
